# Supplementary material for: Development of a computer-aided design software for the quantitative evaluation of aesthetic damage
Source: PLoS One. 2019 Dec 18;14(12):e0226322. doi: 10.1371/journal.pone.0226322 (PMC6919621; doi:10.1371/journal.pone.0226322)
Supplement: S1 Code site — 1. (ZIP) [file pone.0226322.s001.zip › [EESC_jr][Projeto]Relatorio_07_21/reports/templates_zip/gentelella-master/vendors/jszip/documentation/examples/read-local-file-api.html]

---
title: "Reading a local file with the File API"
layout: default
section: example
---

### Choose the local(s) zip file(s)

Note : your browser will process the zip file, don't choose a file too big !

  

You will need a recent browser to use this demo :(

### Content :
